# The roles of maspin expression in gastric cancer: a meta- and bioinformatics analysis

**DOI:** 10.18632/oncotarget.20192

**Published:** 2017-08-11

**Authors:** Hua-Chuan Zheng, Bao-Cheng Gong

**Affiliations:** ^1^ Department of Experimental Oncology and Animal Center, Shengjing Hospital of China Medical University, Shenyang 110004, China

**Keywords:** maspin, gastric cancer, meta analysis, bioinformatics analysis

## Abstract

Maspin is a mammary serine protease inhibitor that is encoded by human *SERPINB5* gene, and inhibits invasion and metastasis of cancer cells as a tumor suppressor. We performed a systematic meta- and bioinformatics analysis through multiple online databases up to Feb 10, 2017. We found down-regulated maspin expression in gastric cancer, compared with normal mucosa and dysplasia (*p* < 0.05). Maspin expression was negatively correlated with depth of invasion, TNM staging and dedifferentiation of gastric cancer (*p* < 0.05). Nuclear maspin expression was higher in intestinal- than diffuse-type carcinoma (*p* < 0.05). An inverse association between maspin expression and unfavorable overall survival was found in patients with gastric cancer (*p* < 0.005). According to bioinformatics databases, *SERPINB5* mRNA expression was higher in gastric cancer than normal tissues (*p* < 0.05), and negatively correlated with depth of invasion, TNM staging and dedifferentiation of gastric cancer (*p* < 0.05). According to KM plotter, we found that a higher *SERPINB5* expression was positively correlated with overall and progression-free survival rates of all cancer patients, even stratified by aggressive parameters (*p* < 0.05). These findings indicated that maspin expression might be employed as a potential marker to indicate gastric carcinogenesis, subsequent progression, and even prognosis.

## INTRODUCTION

Maspin is a mammary serine protease inhibitor that is encoded by human *SERPINB5* gene, and inhibits invasion and metastasis of cancer cells [1, 2]. *SERPINB5* has been identified as a type II tumor suppressor gene in normal mammary epithelial cells by subtractive hybridization, and is located on human chromosome 18q21.3-q23 along with other serpin genes, such as squamous cell carcinoma antigens 1 and 2, PAI-2 and headpin [3, 4]. Maspin is a cytosolic, cell surface-associated, and secretory protein with a reactive center loop that is incompatible with protease inhibition. Maspin has been found to inhibit angiogenesis by stopping the migration, mitogenesis and tube formation of endothelial cells, and to enhance apoptotic sensitivity of cancer cells to extracellular and intracellular stimuli through mitochondria pathway. Maspin retarded Ca^2+^ reduction-induced detachment via a novel interaction with the urokinase-type plasminogen activator/plasminogen [5], and acted as a molecular bridge between the plasminogen activator system and β1 integrin that facilitated cell adhesion in mammary epithelial cells [6]. Odero-Marah et al. [7] found that maspin might inhibit cell motility by suppressing Rac1 and PAK1 activity, and promote cell adhesion via PI3K/ERK pathway. Khalkhali-Ellis et al. [8] reported that secretory maspin could deposit in the extracellular milieu and be incorporated into the matrix for tissue remodeling to suppress invasion. Tamazato et al. [9] demonstrated that EGFR signaling promoted maspin phosphorylation and nuclear localization, where it inhibited gene transcription directly or via histone deacetylase 1, including *CSF-1*, *Bax*, *cytokeratin 18*, and *p21* [10–12].

According to the review [13], maspin expression was down-regulated in melanoma, breast, prostate and gastric cancers, but up-regulated in pancreatic, gallbladder, colorectal, and thyroid cancers, suggesting that maspin might play different roles in various kinds of cancers. *SERPINB5* haploinsufficiency lead to hyperplastic lesions in prostate, and a high sceptibility to hepatocellular carcinoma [14, 15]. Homozygous loss of *SERPINB5* was lethal at the periimplantation stage, due to visceral endoderm dysfunction by reducing cell proliferation and adhesion, thereby controlling early embryonic development [16]. In vial knockout mice, *SERPINB5* deficiency was associated with a reduction in maximum body weight and a variety of context-dependent epithelial abnormalities, such as pulmonary adenocarcinoma, myoepithelial hyperplasia of the mammary gland, hyperplasia of luminal cells of dorsolateral and anterior prostate, and atrophy of luminal cells of ventral prostate and stratum spinosum of epidermis [17].

Since its discovery in 1994, the number of the articles about maspin was increased to 442 in Pubmed database until Feb 10th 2017. The investigators concluded that pattern and level of maspin expression had cell-specific characteristics in malignancies, and closely correlated with its complicated regulators [18–21]. The nuclear or cytoplasmic distribution of maspin has different clinicopathological and prognostic significances in cancers [22–24, 27], even gastric cancer [25–47]. Therefore, we performed a meta and bioinformatics analysis to clarify the roles of maspin expression in gastric cancers.

## RESULTS

### Characteristics of eligible studies

**Figure 1:**
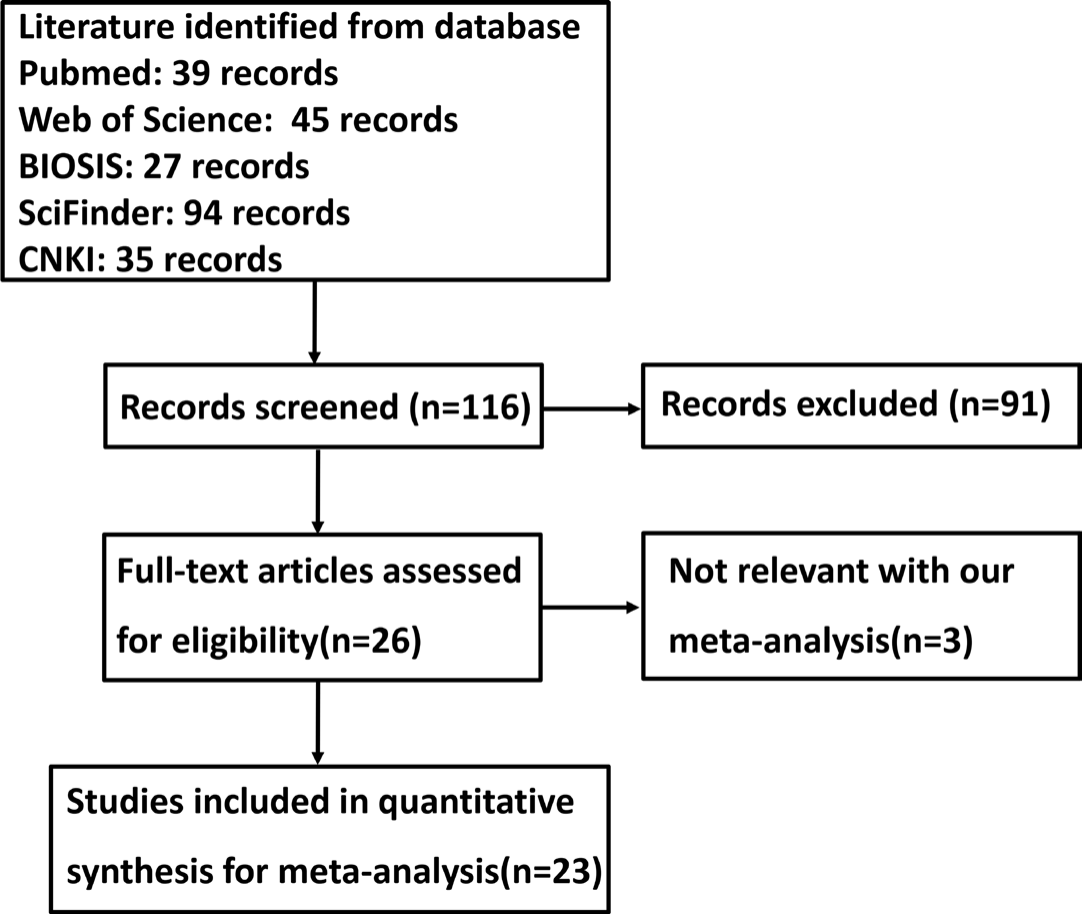
Flow diagram of the selection process in this meta-analysis

Figure 1 is a flow diagram of paper selection for our meta-analysis. As shown in Table 1, a total of 23 articles about the relationship between maspin protein expression and cancer risk, clinicopathological and prognostic parameters of gastric cancer were retrieved for our meta-analysis from PubMed, Web of Science, BIOSIS, SciFinder and CNKI (Chinese). Only 15 articles contained the samples of normal gastric mucosa [27, 32–34, 37–47] and 6 did gastric precancerous lesion-dysplasia [25, 27, 38, 44, 46, 47]. There appeared the comparison between maspin expression and clinicopathological characteristics of gastric cancer in 19 studies, including sex, depth of invasion, lymph node metastasis, TNM staging and Lauren’s classification. Finally, the authors discussed the prognostic significance of maspin expression in 3 articles [33, 35, 36]. There were three articles to compare nuclear or cytoplasmic maspin expression with clinicopathogical features of gastric cancer [27, 29, 36].

**Table 1:** Main characteristics of eligible studies

First author	Year	Country	Ethnicity	Antibody source	Cases	Ctronl	Risk to cancer	Outcome	Quality
Ito R	2004	Japan	Asian	Novo	100		Up		8
Terashima M	2005	Japan	Asian	Pharm	78				8
Yu M	2007	Japan	Asian	Novo	237	23	Up	Neg	9
Zheng HC	2008	Japan	Asian	Novo					8
Gurzu S	2014	Romania	Romanian	Novo	191				8
Gurzu S	2016	Romania	Romanian	Novo	180				8
Kim SM	2005	Korea	Asian	Pharm	62	62	Up		9
Kim YJ	2008	Korea	Asian	BD	109				8
Lee DY	2008	Korea	Asian	Cayman	152	152	Down	Pos	8
Son HJ	2002	Korea	Asian	Pharm	30	26	Up		7
Lei KF	2012	China	Asian	Novo	120			Neg	8
Lei KF	2012	China	Asian	Novo	127				8
Li JJ	2004	China	Asian	Novo	39	39	Up		8
Wang MC	2004	China	Asian	Novo	113	182	Down		8
Bai YX	2007	China	Asian	Maxin	61	10	Down		7
Chen AJ	2009	China	Asian	Neomarker	60	20	Down		7
Cheng SH	2012	China	Asian	Santa	63	20	Down		7
Deng W	2006	China	Asian	Neomarker	60	20	Down		7
Gao P	2007	China	Asian	Neomarker	80	20	Down		7
He Y	2007	China	Asian	Neomarker	172	24	Down		7
Liang QL	2007	China	Asian	Neomarker	102	102	Down		8
Zhang LM	2005	China	Asian	Neomarker	137	54	Down		8
Zhang LP	2012	China	Asian	Neomarker	79	65	Down		8

Note: up, up-regulated; down, down-regulated; Pos, positive correlation; Neg, negative correlation.

### Association between maspin expression and cancer susceptibility of gastric mucosa or dysplasia

**Figure 2:**
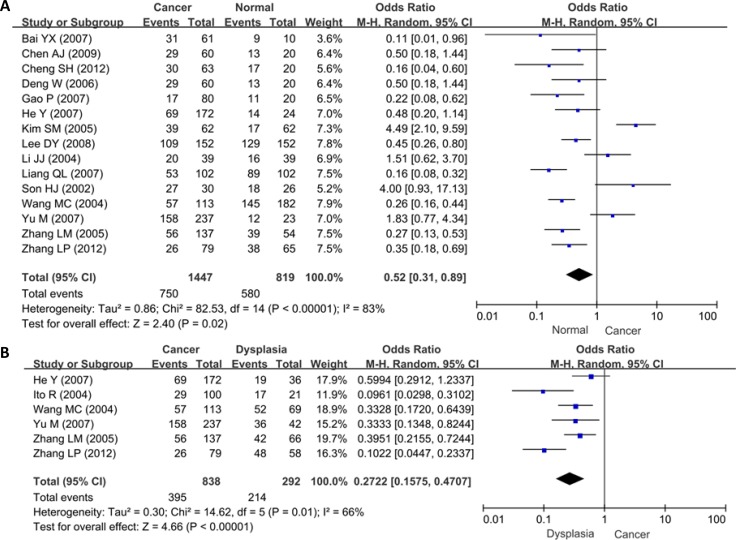

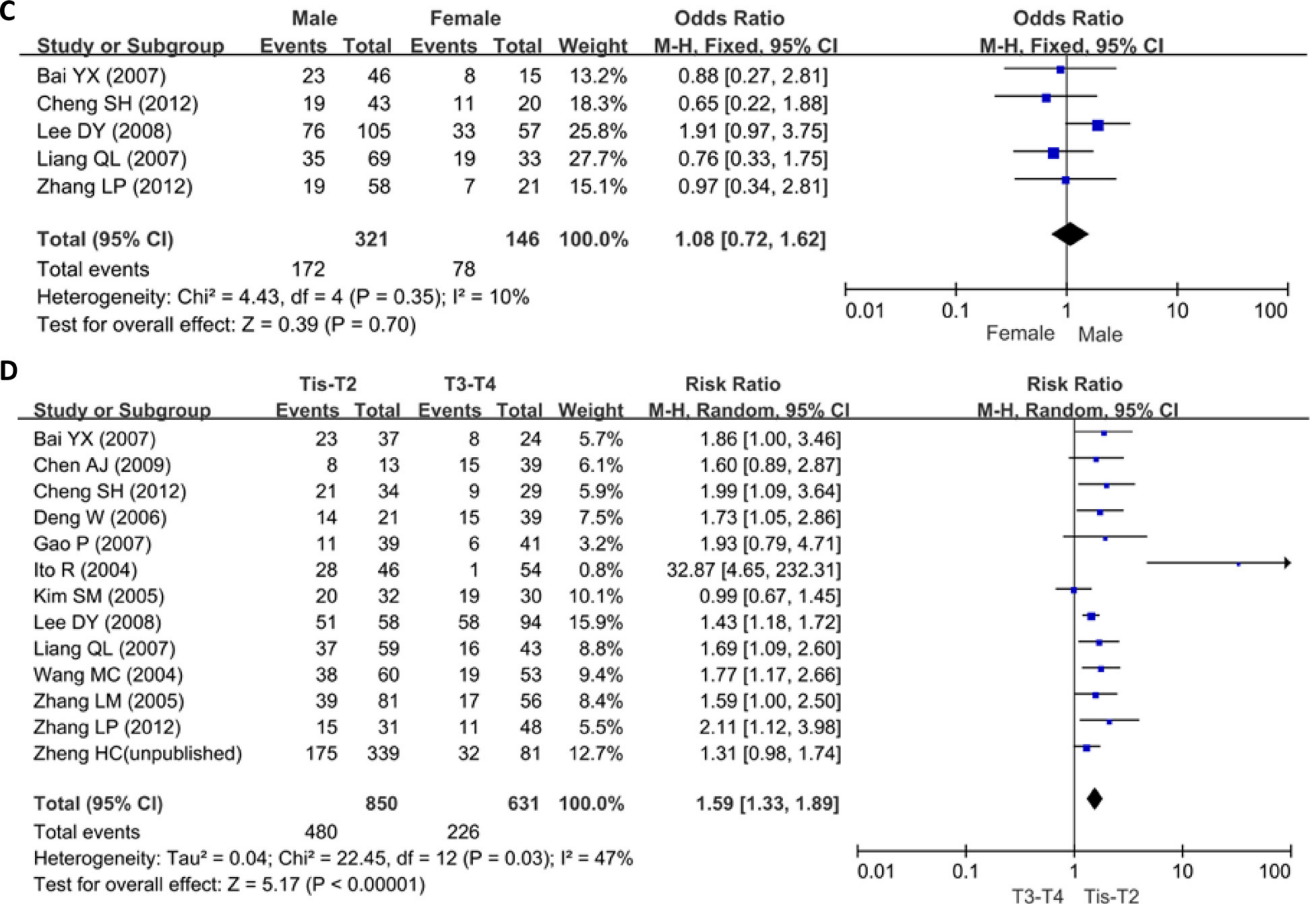

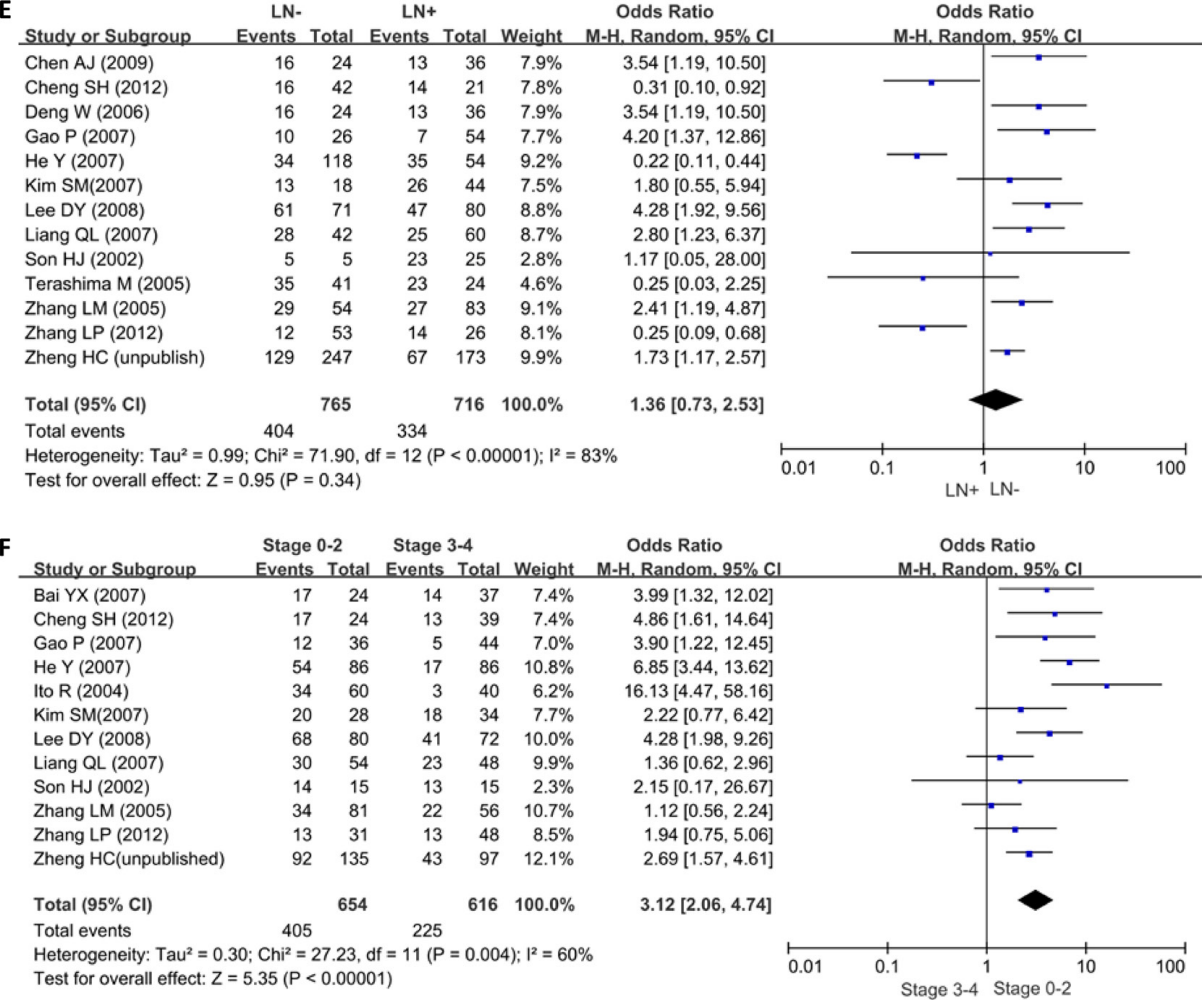

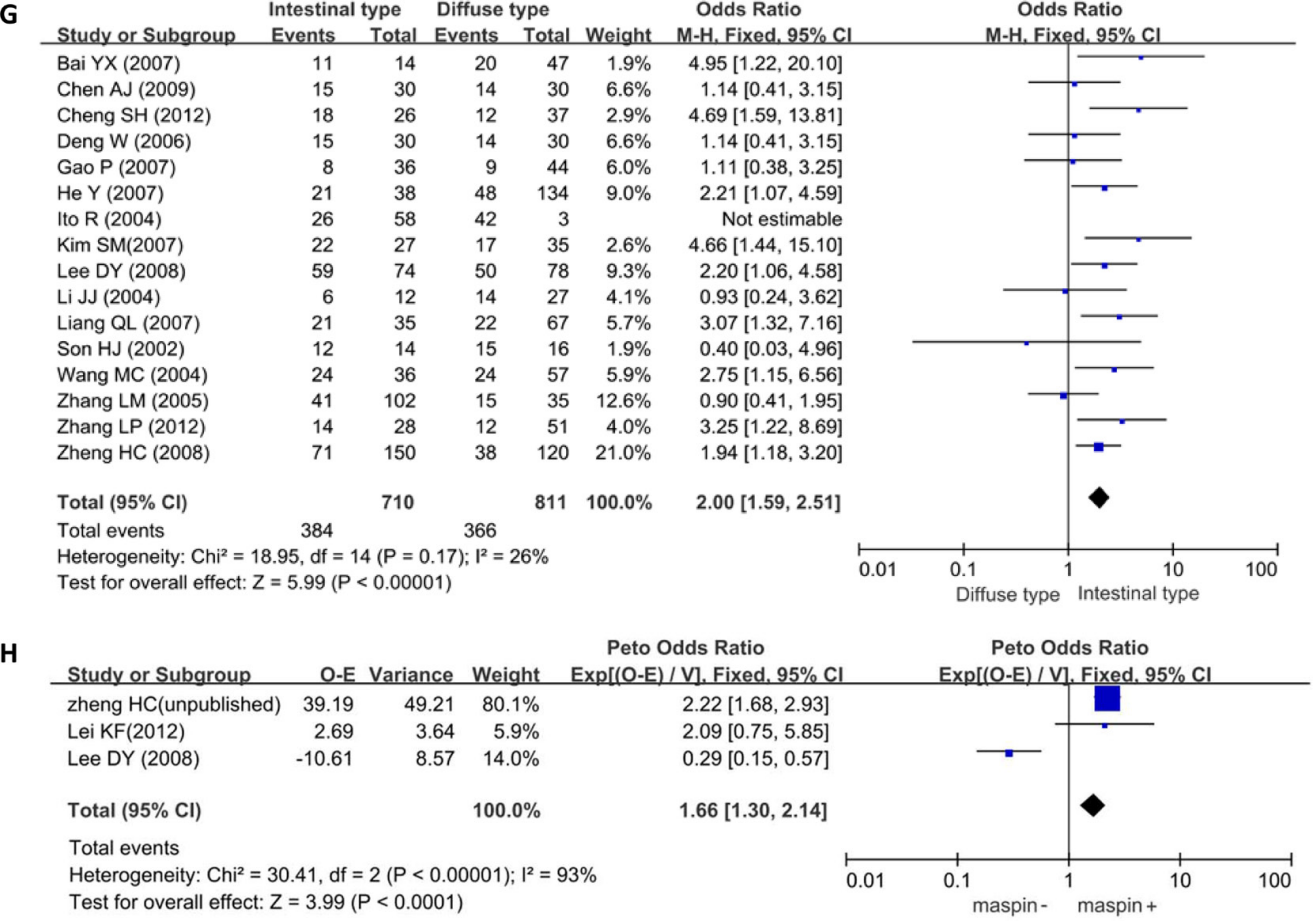
Forest plot for the relationship between maspin expression and clinicopathological parameters of gastric cancer (**A**) gastric carcinogenesis (cancer vs normal mucosa); (**B**) gastric carcinogenesis (cancer vs dysplasia); (**C**) correlation between sex and maspin expression (male vs female); (**D**) correlation between depth of invasion and maspin expression (T_is–2_ vs T_3–4_); (**E**) correlation between lymph node metastasis (LN) and maspin expression (LN- vs LN+); (**F**) correlation between TNM staging and maspin expression (0–II vs III–IV); (**G**) correlation between differentiation and maspin (intestinal-type vs diffuse-type). (**H**) correlation between survival rate and maspin expression.

We analyzed the difference in maspin expression between gastric mucosa and cancer in 15 studies with 1447 cancers and 819 controls. As a result, we found down-regulated maspin expression in gastric cancer, compared with normal mucosa (*p* = 0.02, Figure 2A). Additionally, the same trend was observed using 838 cancers and 292 dysplasia (*p* < 0.00001, Figure 2B).

### Association between maspin expression and clinicopathological parameters of gastric cancer

As shown in Figure 2C, there was no difference in maspin expression between male and female patients with gastric cancer (*p* > 0.05). A higher maspin expression was detected in T_is-2_ than T_3–4_ gastric cancer (*p* < 0.00001, Figure 2D). Maspin expression was not related to lymph node metastasis of gastric cancer (*p* > 0.05, Figure 2E). The patients with stage 0–II cancer showed maspin overexpression, compared with those with stage III-IV cancer (*p* < 0.00001, Figure 2F). Maspin protein showed more expression in intestinal- than diffuse-type carcinomas (*p* < 0.00001, Figure 2G).

**Figure 3:**
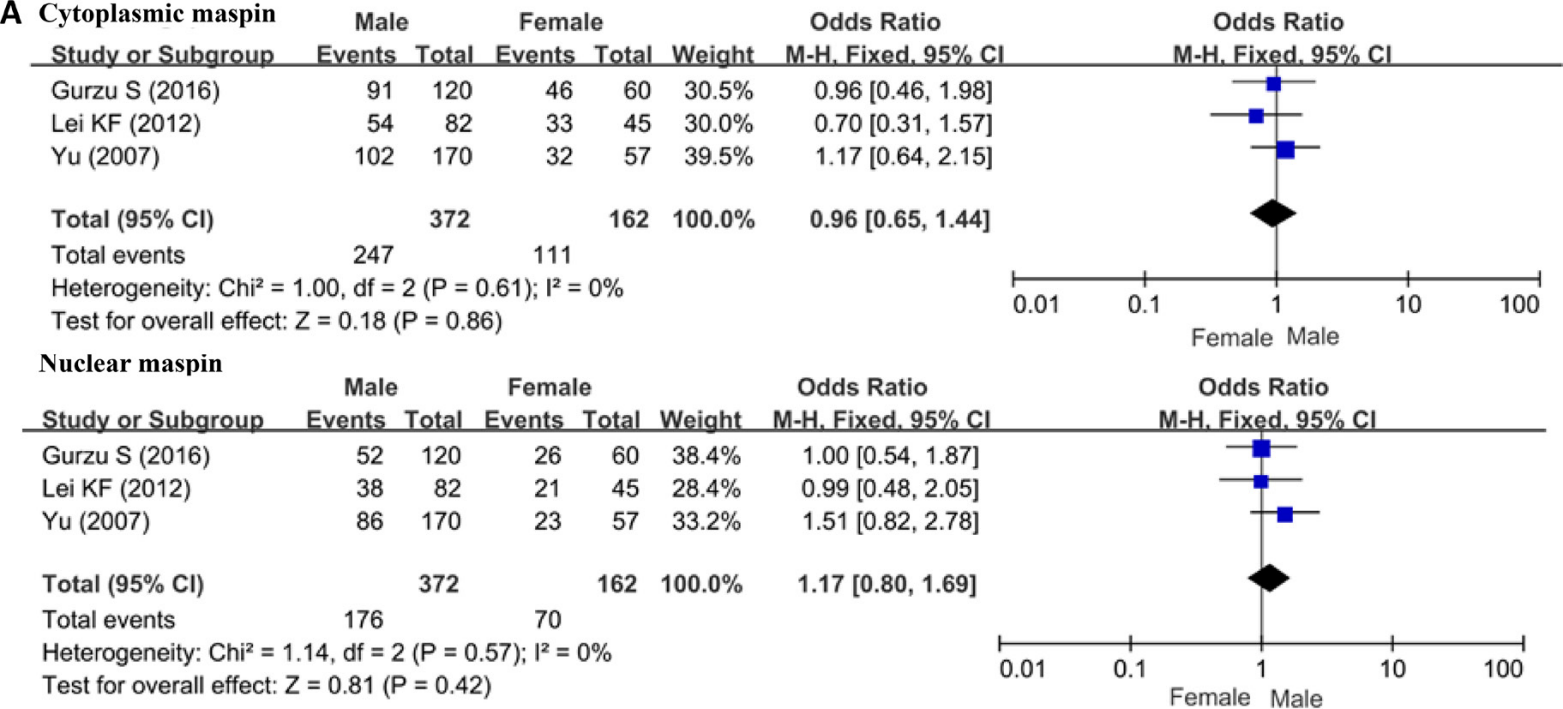

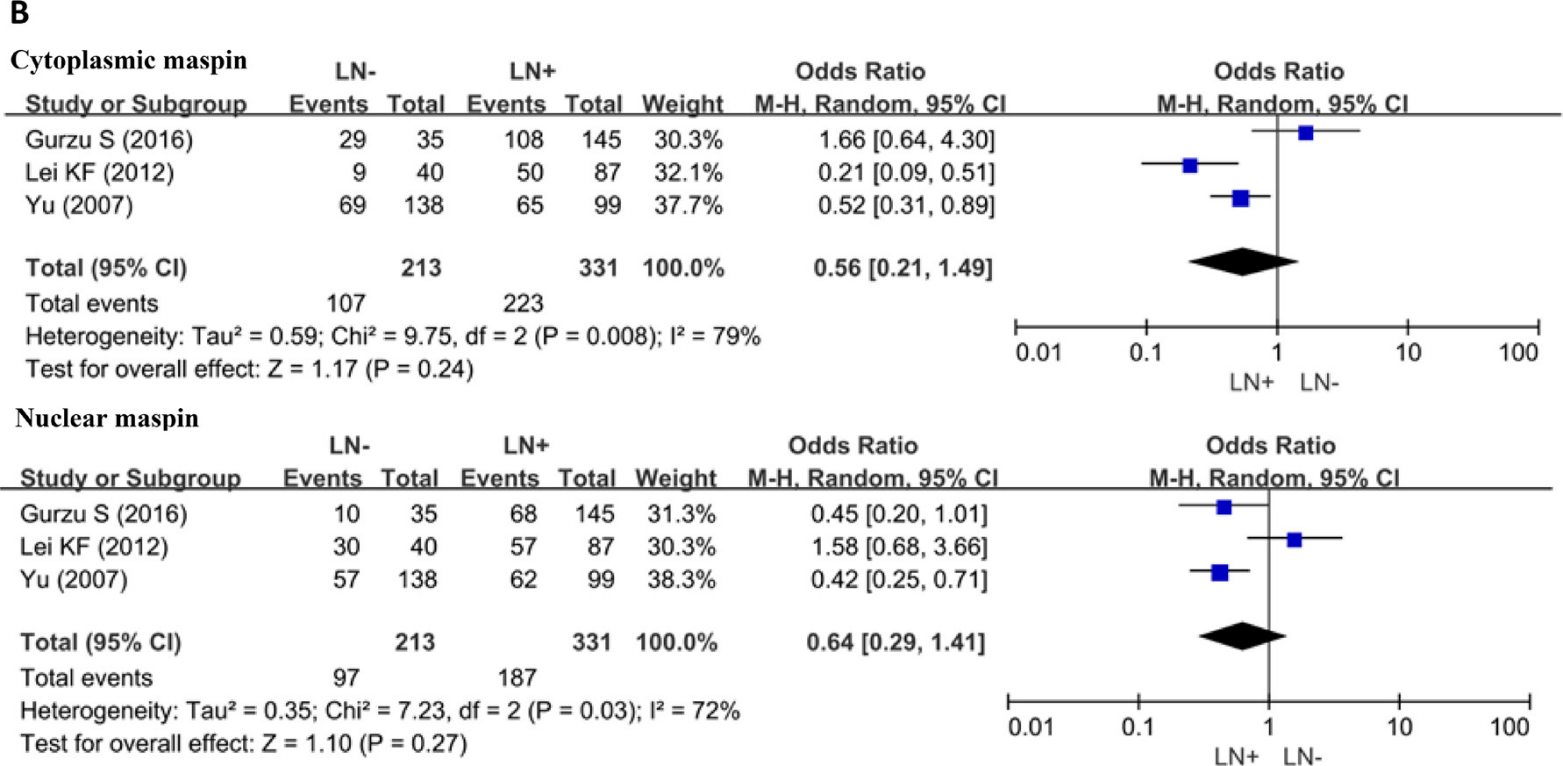

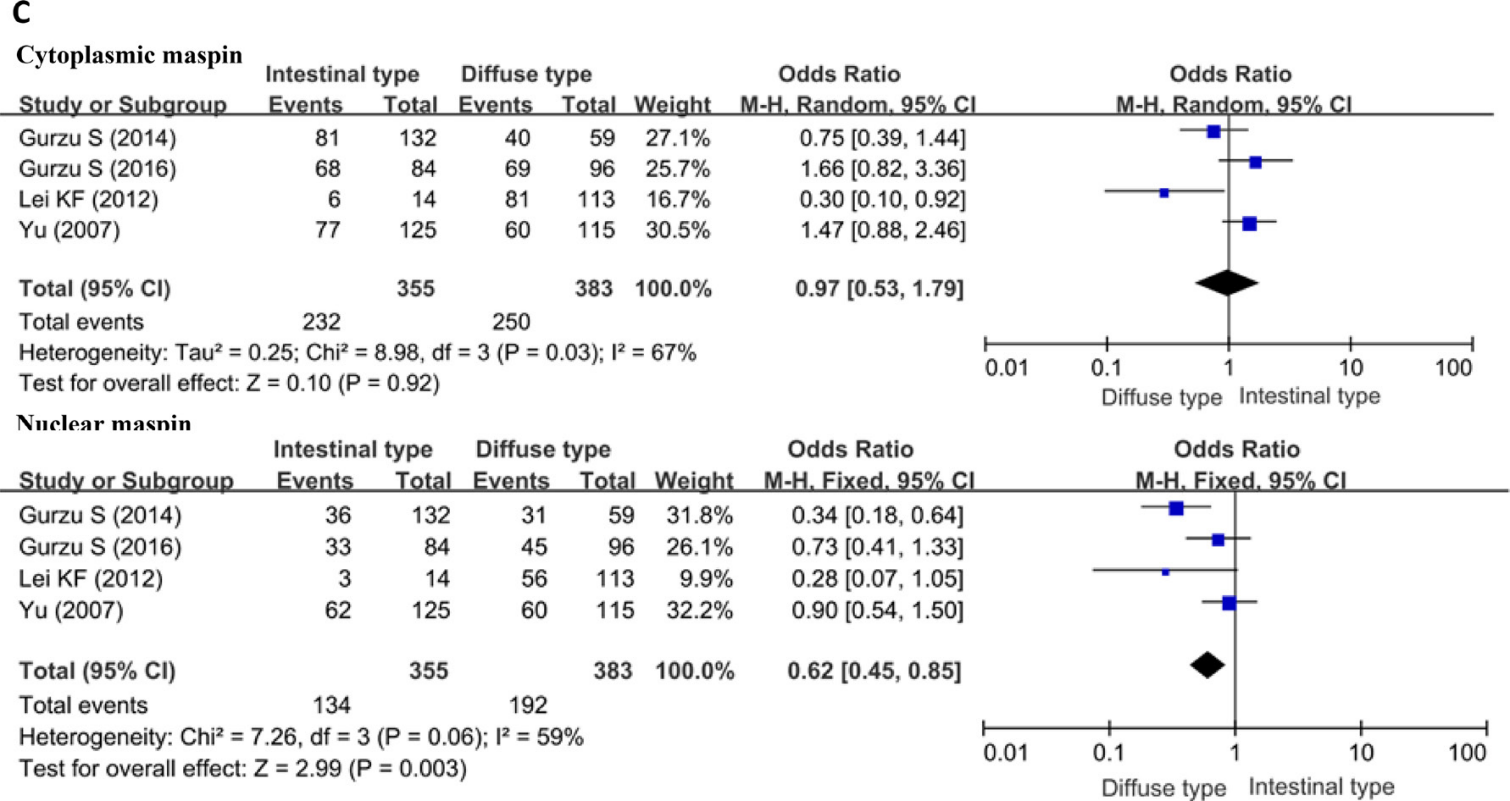
Forest plot for the relationship between cytoplasmic or nuclear maspin expression and clinicopathological parameters of gastric cancer (**A**) correlation between sex and maspin expression (male vs female); (**B**) correlation between lymph node metastasis (LN) and maspin expression (LN− vs LN+); (**C**) correlation between differentiation and maspin (intestinal-type vs diffuse-type).

As indicated in Figure 3A and 3B, neither cytoplasmic nor nuclear maspin expression was correlated with the gender or lymph node metastasis of the patients with gastric cancer (*p* > 0.05). Nuclear maspin expression was higher in diffuse- than intestinal-type carcinomas (*p* < 0.05), but cytoplasmic maspin expression showed no difference between intestinal- and diffuse-type carcinomas (*p* > 0.05, Figure 3C).

### Association between maspin expression and survival rate of gastric cancer

As indicated in Figure 2H, the pooled result from 3 datasets demonstrated a significantly negative association between maspin expression and favorable overall survival in patients with gastric cancer (HR = 1.66, 95% CI: 1.30–2.14, *p* < 0.0001).

### Publication bias

**Figure 4:**
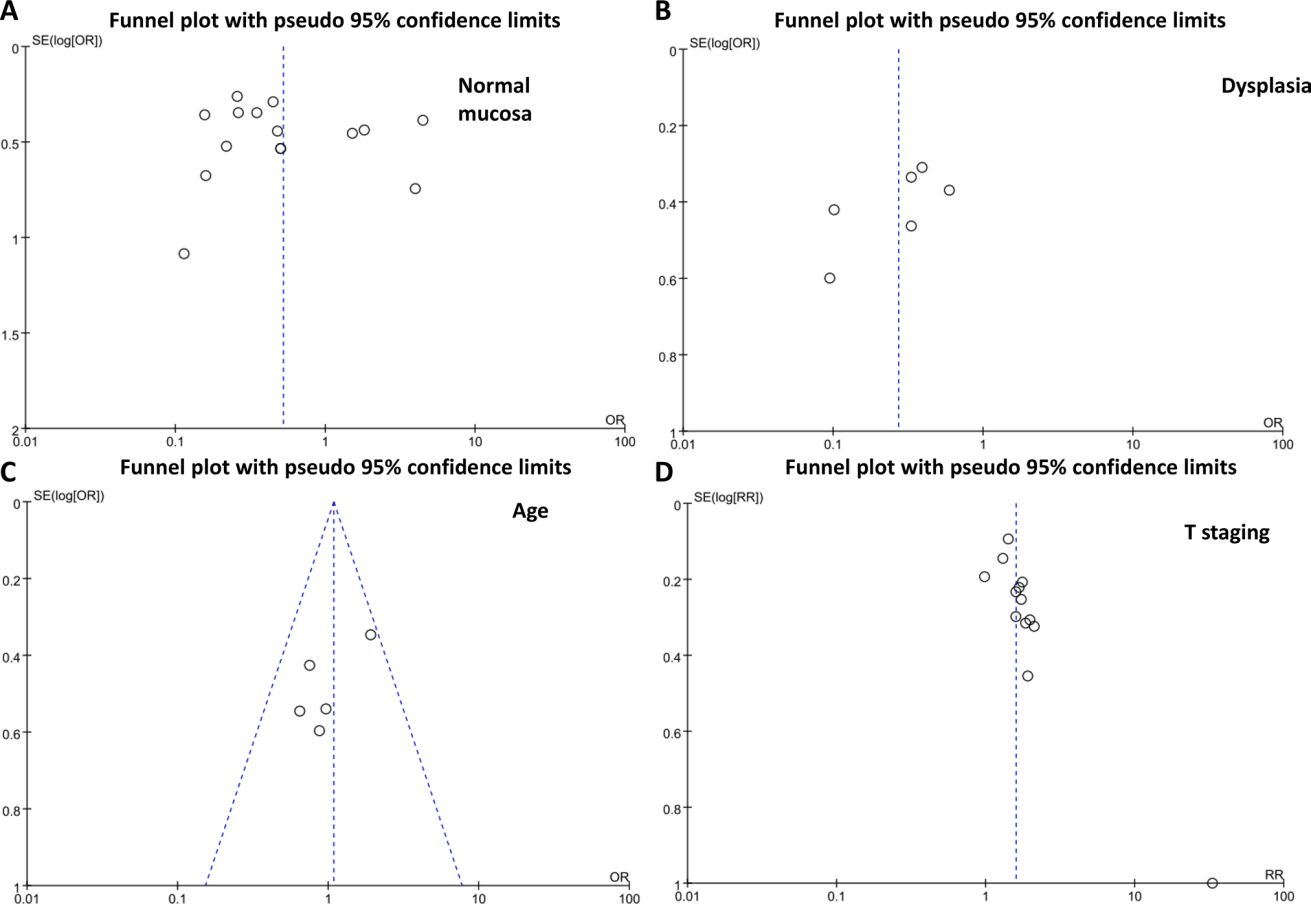

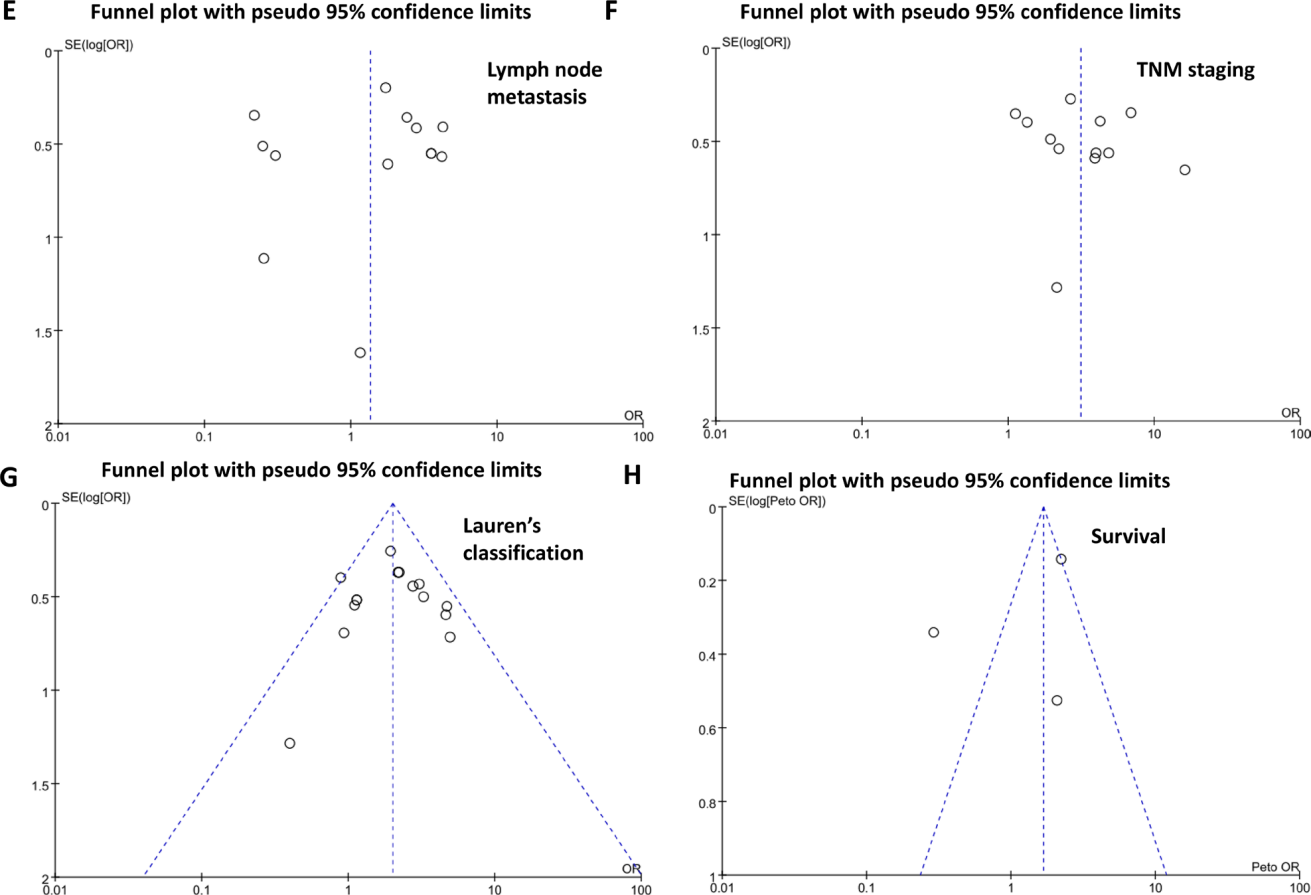

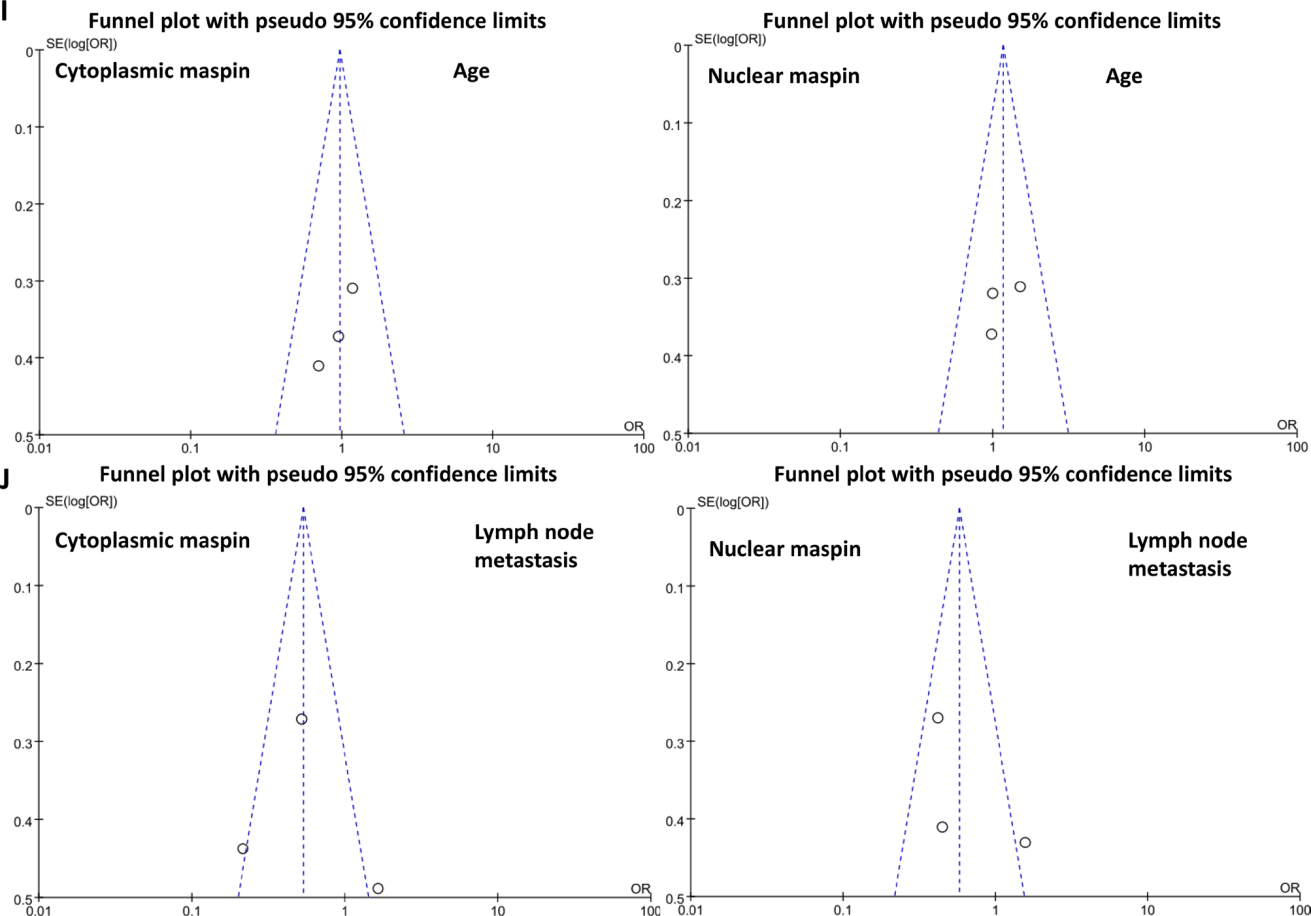

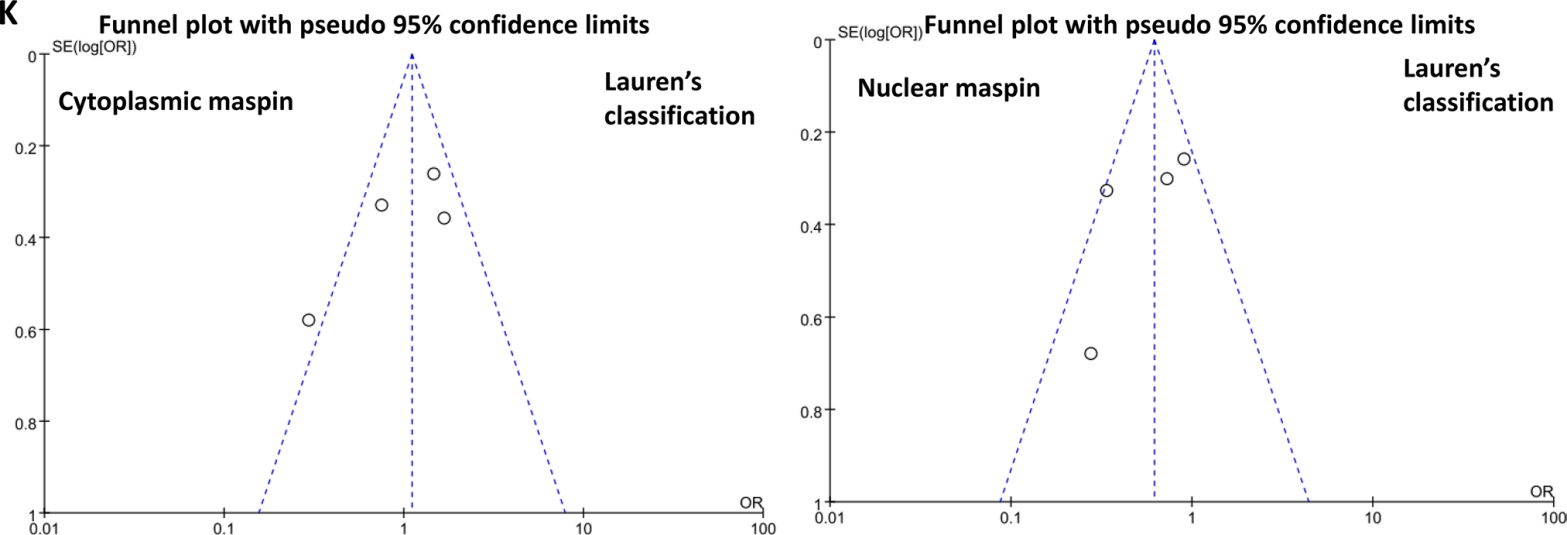
Funnel plot for publication bias test between maspin expression and gastric carcinogenesis or progression The bias was analyzed about risk degrees of maspin expression in gastric mucosa (**A**) and dysplasia (**B**) for gastric carcinogenesis. Additionally, it was tested between maspin expression and clinicopathological features of gastric cancer, including sex (**C**), depth of invasion (**D**), lymph node metastasis (**E**), TNM staging (**F**), and differentiation (**G**) and prognosis (**H**). The bias was analyzed between cytoplasmic or nuclear maspin expression and clinicopathological features of gastric cancer, including age (**I**), lymph node metastasis (**J**), and differentiation (**K**).

The heterogeneity test was performed as shown in Figure 4. Sensitivity analysis was used to evaluate individual study’s influence on the pooled results by deleting one single study each time from pooled analysis. As a result, the prognostic result of maspin expression in Lee’s study had significant effect on the pooled OR. When this study was excluded, the heterogeneity test was significantly reduced (data not shown).

### The clinicopathological and prognostic significances of *SERPINB5* expression in gastric cancer

**Figure 5:**
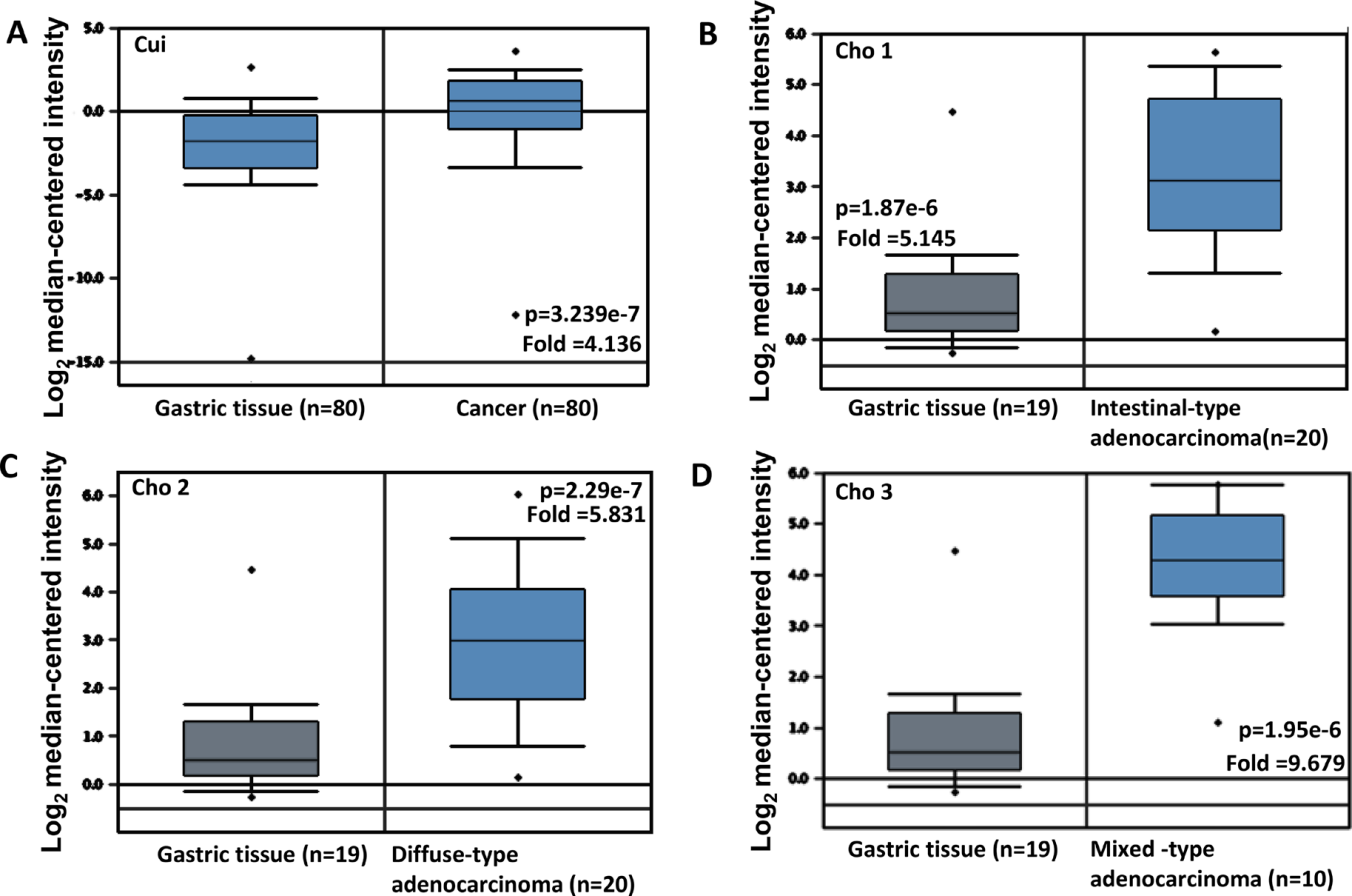

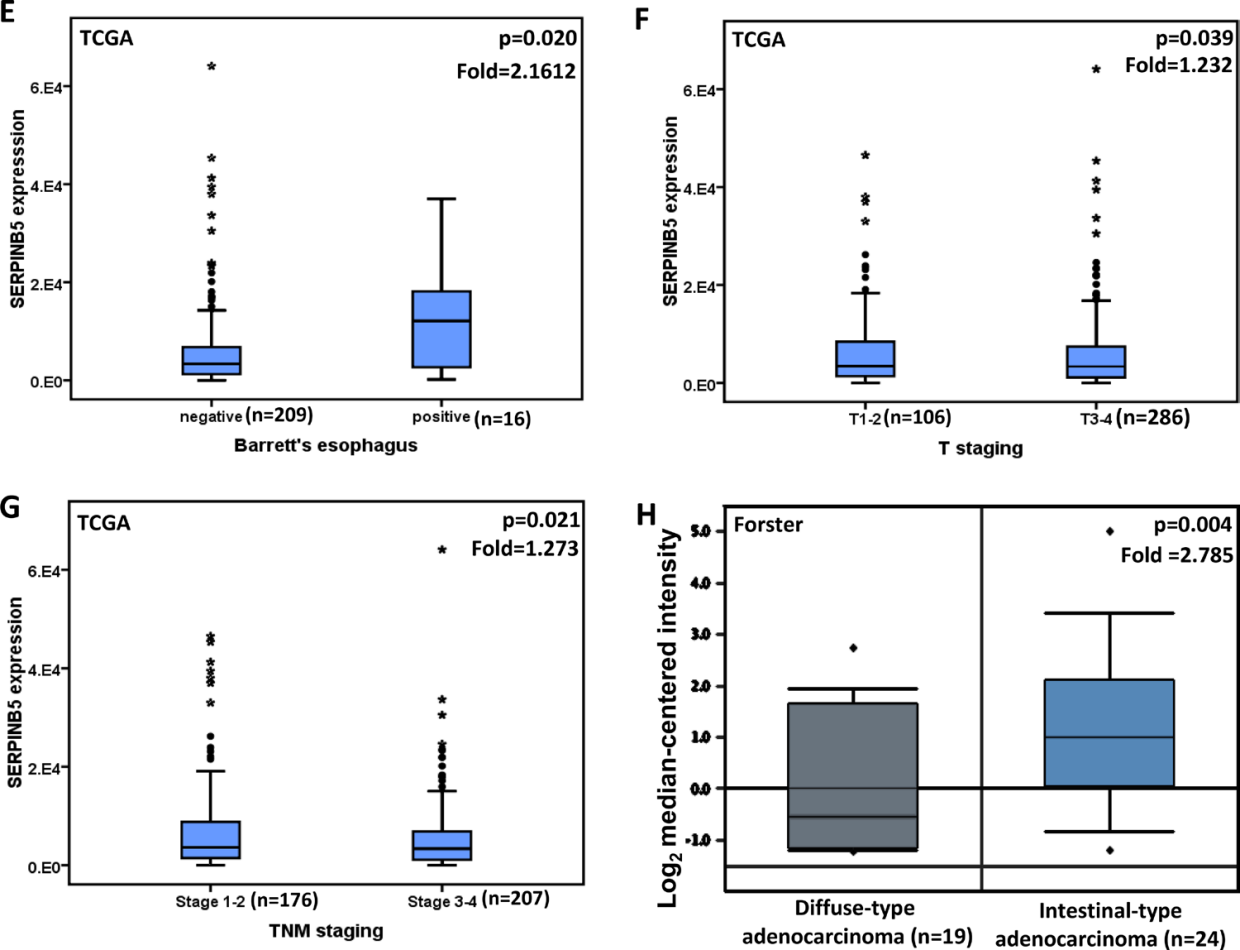
*SERPINB5* mRNA expression in gastric carcinogenesis and subsequent progression Cui's and Cho’s datasets were employed for bioinformatics analysis to analyze *SERPINB5* mRNA expression during gastric carcinogenesis. A higher maspin expression was detectable in gastric cancer than that in normal gastric mucosa ((**A**), *p* < 0.05), even stratified into intestinal- (**B**), diffuse- (**C**), and mixed-type (**D**) carcinomas by Lauren’s classification. TCGA database shows that *SERPINB5* was more expressed in gastric cancer with than without Barrett’s esophagus ((**E**), *p* < 0.05). *SERPINB5* expression was negatively correlated with T staging (**F**) and TNM staging (**G**) of gastric cancers (*p* < 0.05). According to Forester’s database, there appeared a higher *SERPINB5* expression in gastric intestinal- than diffuse- type carcinomas ((**H**), *p* < 0.05).

We used TCGA's, Cui's and Cho's datasets to perform bioinformatics analysis, and found that SERPINB5 mRNA expression was lower in gastric normal than cancer tissues, even stratified into intestinal-, diffuse- and mixed-type carcinomas (Figure 5A–5D, *p* < 0.05). In TCGA data, *SERPINB5* expression was higher in gastric cancers with than without Barret’s esophagus (*p* < 0.05, Figure 5E). It was negatively correlated with depth of invasion and TNM staging of gastric cancer (*p* < 0.05, Figure 5F and 5G). Forester's data showed a higher *SERPINB5* mRNA expression in intestinal-type than diffuse-type carcinomas (*p* < 0.05, Figure 5H).

**Figure 6:**
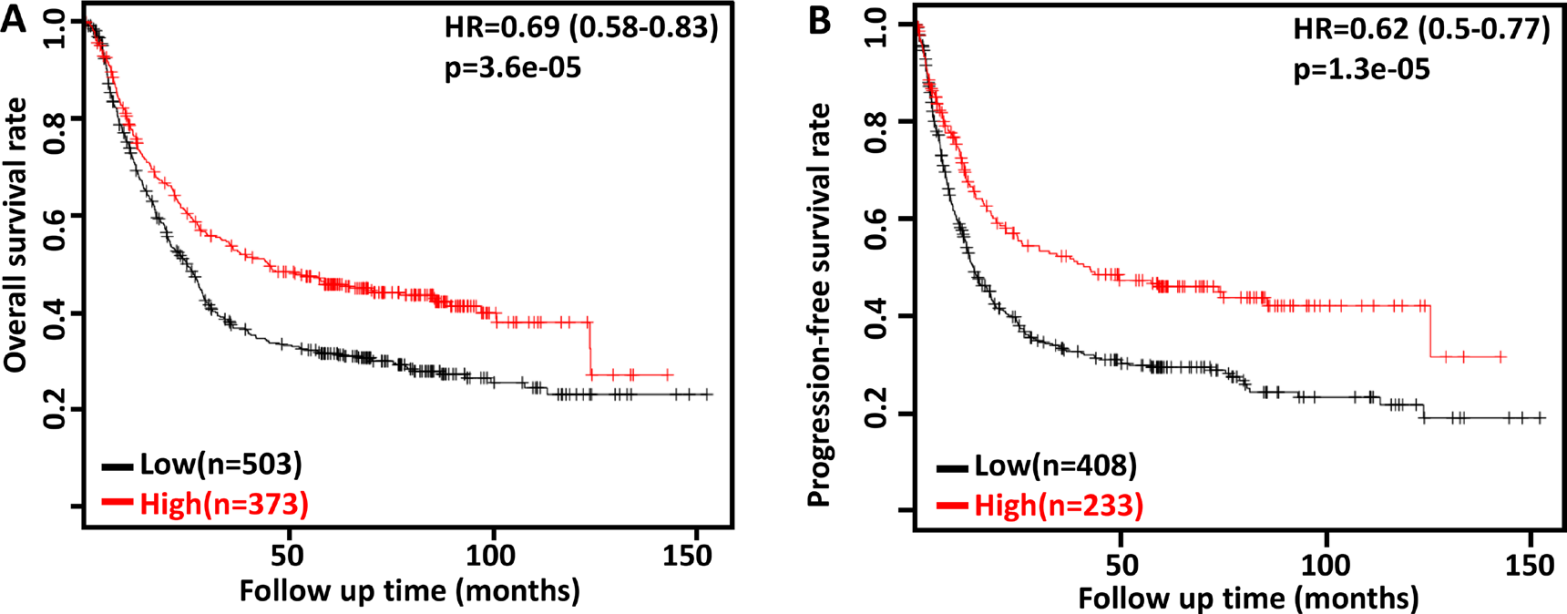
The prognostic significance of *SERPINB5* mRNA in the patients with gastric cancer According to the data from KM plotter, *SERPINB5* mRNA expression was positively related to both overall (**A**) and progression-free (**B**) survival rates of the patients with gastric cancer. HR, hazard ratio.

According to Kaplan-Meier plotter, we found that a higher *SERPINB5* mRNA expression was positively correlated with overall and progression-free survival rates of all cancer patients, the patients with surgery alone or 5-FU-based adjuvant treatment, the patients with Her2- or Her2+, the patients with no distant metastasis, no lymph node metastasis, lymph node metastasis, N_1_ or N_2_ status, and female or male patients (*p* < 0.05, Figure 6A and 6B and Table 2). Stage I–IV cancer patients with high *SERPINB5* mRNA expression showed a long overall survival time than those with its low expression (*p* < 0.05), while it was the same for progression-free survival in the patients with stage II and III cancer (*p* < 0.05). There appeared a positive relationship between *SERPINB5* mRNA expression and the overall survival rate of the intestinal- and diffuse-type carcinoma patients (*p* < 0.05), whereas the same correlation between *SERPINB5* mRNA expression and progression-free survival was observed in diffuse- and mixed-type carcinoma patients (*p* < 0.05). The overall survival rate of the patient with T_2_ or T_3_ cancer was positively linked to *SERPINB5* mRNA expression (*p* < 0.05). Positive association between *SERPINB5* mRNA expression and progression-free prognosis was observed in T_2_ cancer patients (*p* < 0.05).

**Table 2:** The prognostic significance of *SERPINB5* mRNA in gastric cancer by Kaplan-Meier plotter

Clinicopathological features	Overall survival		Progression-free survival	
	Hazard ratio	*p*	Hazard ratio	*P*
Sex				
Female	0.53 (0.34–0.83)	0.004	0.5 (0.31–0.81)	0.0042
Male	0.63 (0.51–0.77)	1.4e-05	0.62 (0.49–0.78)	6.7e-05
T				
2	0.52 (0.34–0.79)	0.002	0.51 (0.33–0.78)	0.0019
3	0.62 (0.41–0.95)	0.026	0.74 (0.53–1.04)	0.086
4	0.55 (0.21–1.46)	0.23	0.64 (0.29–1.4)	0.26
N				
0	0.41 (0.17–0.95)	0.032	0.4 (0.17–1.02)	0.048
1–3	0.58 (0.44–0.77)	9.7e-05	0.59 (0.45–0.77)	7.6e-05
1	0.46 (0.29–0.72)	0.00047	0.47 (0.31–0.72)	0.00038
2	0.68 (0.43–1.09)	0.11	0.69 (0.44–1.08)	0.1
3	0.62 (0.36–1.06)	0.079	0.67 (0.39–1.16)	0.15
M				
0	0.59 (0.44–0.78)	0.00027	0.6 (0.45–0.79)	0.00025
1	1.79 (0.95–3.35)	0.067	1.71 (0.95–3.1)	0.072
TNM staging				
I	0.25 (0.07–0.88)	0.02	0.31 (0.09–1.13)	0.062
II	0.28 (0.13–0.58)	3e-04	0.33 (0.16–0.68)	0.0014
III	0.59 (0.44–0.8)	0.00057	0.59 (0.38–0.93)	0.021
IV	0.64 (0.42–0.99)	0.042	0.8 (0.52–1.23)	0.31
Perforation				
-	0.75 (0.47–1.18)	0.21	0.73 (0.47–1.13)	0.15
Differentiation				
Well-differentiated	0.46 (0.18–1.14)	0.086		
Moderately-differentiated	1.39 (0.73–2.66)	0.32	1.4 (0.75–2.61)	0.29
Poorly-differentiated	0.72 (0.45–1.14)	0.16	0.72 (0.43–1.21)	0.22
Lauren’s classification				
Intestinal-type	0.55 (0.4–0.75)	0.00013	0.78 (0.55–1.11)	0.17
Diffuse-type	0.53 (0.37–0.77)	0.00054	0.45 (0.3–0.68)	9e-05
Mixed-type	0.38 (0.13–1.07)	0.058	0.2 (0.06–0.66)	0.0039
Her2 positivity				
−	0.63 (0.49–0.81)	0.00039	0.6 (0.46–0.82)	0.00081
+	0.62 (0.48–0.8)	3e-04	0.54 (0.39–0.75)	0.00016
Treatment				
Surgery alone	0.65 (0.48–0.88)	0.0044	0.62 (0.46–0.84)	0.0015
5-FU-based adjuvant	1.63 (1.11–2.4)	0.013	1.66 (1.13–2.45)	0.0091
Other adjuvant	0.46 (0.19–1.12)	0.079	0.5 (0.23–1.1)	0.079

## DISCUSSION

Metastasis is the most critical impediment for the survival of cancer patients. Maspin reintroduction was found to reverse epithelial-to-mesenchymal transition of prostate cancer cells by inhibiting HDAC1 activity and suppressing TGF-β/β-catenin /E-cadherin pathway [48, 49]. Lee et al. [50] demonstrated that maspin increased Ku70 acetylation by inhibiting HDAC1, and subsequently caused Bax-mediated cell death by dissociation of Bax from Ku70. Endsley et al. [51] found that maspin mediated the molecular bridge between the plasminogen activator system and β1 integrin that facilitated cell adhesion in mammary epithelial cell. To investigate the clinicopathological and prognostic significances of maspin expression, we analyzed 23 studies, which met specific inclusion criteria and had moderate to high quality according to their NOS scores. Additionally, we also added our unpublished data about maspin expression.

According to the literature [52], precancerous lesions appear from gastric epithelium to adenocarcinoma, including adenomatous, regenerative, crysptal or globoid dysplasia. Consistent with the data about breast, colonic, bladder and gastric cancers [15, 27], we found down-regulated maspin expression in gastric cancer, compared with gastric mucosa or dysplasia in the present study, suggesting that maspin hypoexpression contributed to gastric carcinogenesis as a late event. Previously, we performed maspin immunostaining using 2 individual samples of gastric cancer and found its upregulation. The discrepancies might be largely attributable to organ-specificity, criteria for positive staining, statistical analysis and subjects. Moreover, we confirmed the similar maspin expression in gastric cancer cells despite different antibodies from Novocastra and BD Pharmagin used [27]. Another explanation might be disadvantage of tissue microarray: too small sample not enough to represent the overall appearance. If the area of maspin negativity was frequently selected, the positive rate of maspin was decreased.

Although Dabiri et al. [53] found that *SERPINB5* mRNA level was considerably lower in the cancer samples compared with normal breast samples, up-regulated expression of *SERPINB5* mRNA was observed from 2 databases in line with 8-fold increase of *SERPINB5* mRNA in gastric cancer [26]. Lu et al. [54] found that *SERPINB5* mRNA expression was up-regulated in pulmonary adenocarcinoma samples in comparison to the adjacent normal tissues. This is not surprising since mRNA levels do not usually predict the corresponding protein levels because it takes a long distance from mRNA to functional protein by translation and posttranslational modification.

Previously, we found that the high expression of cytoplasmic and nuclear maspin was positively correlated with aggressive parameters of gastric cancer, including invasion, metastasis and tumor size [27]. Pföhler el al. [55] found that maspin expression in the invasive margin of primary melanomas might reflect aggressive phenotypes, including Clark level, tumor thickness and disease stage. In contrast, our findings showed maspin expression was inversely linked to depth of invasion, TNM staging and dedifferentiation of gastric cancer regardless of its mRNA and protein, indicating that its hypoexpression was involved in invasion and progression of gastric cancer, in agreement with the reports about breast, prostatic, colonic, bladder, and cervical cancers [15, 27]. Here, nuclear maspin immunoreactivity also appeared positive association with differentiation of gastric cancer, which might be attributed to the selection bias because only 3 studies were involved in our analysis. Taken together, we concluded that maspin expression loss might be employed as a potential biomarker for aggressiveness of gastric cancers.

Reportedly, maspin overexpression is associated with better overall survival in esophageal and oral squamous cell carcinoma [56, 57]. Shift from cytoplasmic to nuclear maspin expression was correlated with shorter overall survival in node-negative colorectal cancer and lung cancer [58, 24]. However, Snoeren et al. [59] found that maspin expression was a marker for early recurrence in primary stage III and IV colorectal cancer, and its overexpression was correlated with poor outcome after cancer spread to the local lymph nodes. Our meta-analysis showed that maspin expression was positively linked to the worse prognosis of the patients with gastric cancer. Here, our unpublished data mainly determined the final outcome, which included the cases from Yu et al. [27] and Zheng et al. [28]. However, our bioinformatics data indicated that *SERPINB5* mRNA expression was positively associated with overall and progression-free survival rates of the patient with gastric cancer, even stratified by clinicopathological features, opposite with the report about pulmonary adenocarcinoma of Lu et al. [54]. The paradoxical phenomenon might be due to the distinct sensitivity of different methodologies: bioinformatics analysis is based on RNA sequencing, but Lu’s experiment on RT-PCR.

Several limitations should be noted in our meta-analysis. Firstly, the potential publication bias stems from published results being predominantly positive. Secondly, patient populations in our study are limited because the patients come only from Asia and Romania. Thirdly, all of the survival data are extracted from survival curves, which may introduce subjective bias. Fourthly, this small sample size limits the power to detect the associations in some articles. Fifthly, we add more cases of gastric cancer for our analysis, which also increases the possibility of selection bias.

In conclusion, maspin expression underwent a down-regulation in gastric carcinogenesis as a late event, but versa for its mRNA. It was negatively correlated with depth of invasion, TNM staging and dedifferentiation of gastric cancer at both mRNA and protein levels. Maspin expression might be employed as a good potential marker for worse prognosis of gastric cancer patients, while it was the converse for its mRNA.

## MATERIALS AND METHODS

### Identification of eligible studies and data extraction

We performed a publication search using PubMed, Web of Science, BIOSIS and SciFinder updated on Feb 10, 2017. The following search terms were used: (maspin OR SERPINB5 OR PI5) AND (gastric OR stomach) AND (cancer OR carcinoma OR adenocarcinoma). Searching was done without restriction on language or publication years. Inclusion criteria for studies: (1) articles to observe the alteration in maspin expression in gastric cancer by immunohistochemistry; (2) papers to compare maspin expression with pathobiological behaviors and prognosis of gastric cancer by immunohistochemistry. Exclusion criteria included: (1) abstract, comment, review and meeting; (2) duplication of the previous publications; (3) Western blot, RT-PCR, cDNA microarray, or transcriptomic sequencing for maspin expression; (4) lack of sufficient information.

### Data extraction

Based on the inclusion criteria, two reviewers (Zheng HC and Gong BC) independently extracted information from all eligible publications. The following information was included in each study: name of first author, year of publication, country, ethnicity, antibody company, numbers of cases and controls, expression alteration, and follow-up outcome. Regarding survival analysis, we used Engauge Digitizer software to extract data from Kaplan-Meier curves and calculated the Hazard ratios and their corresponding 95% confidence intervals. Any disagreement was resolved through discussion until the two reviewers reached a consensus.

### Quality score assessment

Two reviewers (Zheng HC and Gong BC) independently assessed the quality of the included studies according to Newcastle Ottawa Scale (NOS) (http://www.ohri.ca/programs/clinical_epidemiology/oxford.htm). The scale consists of three components related to sample selection, comparability and ascertainment of outcome.

### Bioinformatics analysis

The individual gene expression level of *SERPINB5* was analyzed using Oncomine (www. oncomine.org), a cancer microarray database and web-based data mining platform for a new discovery from genome-wide expression analyses. We compared the differences in *SERPINB5* mRNA level between gastric normal tissue and cancer. All data were log-transformed, median centered per array, and standard deviation normalized to one per array. The expression (RNA-seqV2) and clinicopathological data of 325 gastric cancer patients were downloaded from the Cancer Genome Atlas (TCGA) database by TCGA-assembler in R software. We integrated the raw data, analyzed *SERPINB5* expression in gastric cancer, and compared it with clinicopathological and prognostic data of the patients with gastric cancer. Additionally, the prognostic significance of *SERPINB5* mRNA was also analyzed using Kaplan-Meier plotter (http://kmplot.com).

### Statistics analysis

HWE was evaluated using Chi-square test in control groups of each study. Strength of association between maspin expression and cancer risk was assessed by odds ratios with 95% confidence intervals. Statistical significance of the pooled OR was determined by *Z* test. If there was no significant heterogeneity, the fixed effect model (Mantel–Haenszel method) would be employed. Otherwise, the random effect model (DerSimonian and Laird method) would be used excluding prognostic analysis. Heterogeneity effect was then quantified by *I*^2^ test, which was subdivided into low, moderate and high degrees of heterogeneity according to the cut-off values of 25%, 50% and 75% respectively. Publication bias was evaluated by funnel plot and quantified by Begg’s test and Egger’s test to assess funnel plot asymmetry. Meta-analyses were performed with Revman software 5.3 and data from TCGA database was dealt with SPSS 10.0 software using student *t* test. Two-sided *p* < 0.05 was considered as statistically significant. SPSS 17.0 software was employed to analyze all data.
